# Investigating defensive functioning and alexithymia in substance use disorder patients

**DOI:** 10.1186/s12888-021-03340-w

**Published:** 2021-07-06

**Authors:** Alessandro Taurino, Linda A. Antonucci, Paolo Taurisano, Domenico Laera

**Affiliations:** 1 Department of Education, Psychology, Communication, University of Bari, Palazzo Chiaia-Napolitano, Via Scipione Crisanzio 42, 70122 Bari, Italy; 2 Section of Clinical Psychology and Neuropsychology, Department of Basic Medical Sciences, Neuroscience and Sense Organs, University of Bari, Bari, Italy

**Keywords:** Defense mechanisms, Addiction, Alexithymia, Personality

## Abstract

**Background:**

Substance Use Disorder (SUD) causes a great deal of personal suffering for patients. Recent evidence highlights how defenses and emotion regulation may play a crucial part in the onset and development of this disorder.

The aim of this study was to investigate potential differences in the defensive functioning between SUD patients and non-clinical controls. Secondly, we aimed at investigating the relationships between alexithymia and maladaptive/assimilation defenses.

**Methods:**

The authors assessed defensive functioning (Response Evaluation Measure-71, REM-71), personality (MMPI-II), and alexithymia (TAS-20) of 171 SUD patients (17% female; mean age = 36.5), compared to 155 controls. Authors performed a series of ANOVAs to investigate the defensive array in SUD patients compared to that of non-clinical controls. Student t test for indipendent samples was used to compare clinical characteristics between the SUD group and the controls. To investigate the role of single defenses in explaining alexithimia’s subscores, stepwise multiple regression analysis were carried out on socio-demographic characteristics of participants (gender, age, and years of education), with REM-71 defenses as predictors.

**Results:**

SUD patients presented a more maladaptive/assimilation (Factor 1) defensive array (*p* < .001). Among SUD sub-groups, Alcohol Use Disorder patients showed more disfuncional defenses. Factor 1 defenses were related to a worse psychological functioning. In addition, alexyhimia (particularly DIF) was strongly related to Factor 1 defenses, expecially *Projection* (38% of variance explained, β = .270, *p* < .001).

**Conclusion:**

The REM-71 and the TAS-20 might be useful screening instruments among SUD patients.

## Introduction

According to the *Diagnostic and Statistical Manual of Mental Disorders*, *fifth edition* (DSM-5) [1], Substance Use Disorder (SUD) “describes a problematic pattern of using alcohol or another substance that results in impairment in daily life or noticeable distress”. Diagnostic criteria are uniform across different drugs of abuse and include common-sense symptoms such as craving, tolerance, withdrawal, failure to fulfill social obligations, and use despite harms.

SUDs cause a great deal of personal suffering for patients and families, and have devastating psychological, medical, and social effects [2]. Despite the prevalence and the numerous disabling outcomes associated with SUD, its risk and protective factors from both the individual and social perspectives are still unclear. Research has mostly investigated personality traits [3], psychopathology [4], and coping style [5], but recent evidence highlights how defense mechanisms and emotion regulation may play a crucial part in the onset and development of this disorder [6, 7].

First described by Sigmund Freud (1894) and later systematized by Anna Freud (1936), defenses represent involuntary cognitive operations that occur outside of awareness to minimize sudden changes in internal and external environments by modifying the conscious experience of thought, feeling, and emotion [8]. The DSM-5 defines defenses as “mechanisms that mediate the individual’s reaction to emotional conflicts and to external stressor. Some defense mechanisms are invariably maladaptive. Others may be either maladaptive or adaptive, depending on their severity, their inflexibility, and the context in which they occur” [1]. Defenses could be arranged according to the Vaillant continuum ranging from being maladaptive to being adaptive [8].

Findings from several studies suggest that defenses are related to psychological health and well-being, psychiatric symptoms, and some evidence suggests that defenses may antedate symptoms and predispose to psychopathology [9]. Defensive functioning is found to be associated with personality disorders [10], and several emotional problems [11]. Maladaptive defenses are strongly associated with several other indices of poorer mental health and relatedness, encompassing alexithymia [12], insecure attachment [13, 14], distancing/avoidance coping strategies [15], deliberate self-harm [16], and misperception of mortal risks [17]. Adaptive defenses tend to be associated with better health conditions [18].

Among the few studies available on defense functioning in SUDs, substance-dependent individuals use rationalization, projection, denial, and suppression defenses more than healthy individuals [19]. Studies have shown that SUD is associated with more frequent use of immature defense mechanisms such as autistic fantasy, acting out and isolation [5]. Consistently, in young alcohol dependent individuals, acting out was associated with a higher risk of self-injury, while less frequent use of mature defense mechanism seems to be a predictor of suicidal behavior in the same population [20]. Acting out defense has also been previously associated with relapse to alcohol use 12 months after inpatient treatment [21]. Notably, previous studies also highlighted the association between maladaptive defense mechanisms and antisocial behavior in alcohol dependent patients [22]. This is consistent with other studies in which substance dependent patients exhibited a significant association between the use of immature defenses and severity of dependency, dissociative experiences, and alexithymia [23].

Indeed, there is a high prevalence of alexithymia in patients with SUDs [24].

Used for the first time by Sifneos [25] to describe certain clinical characteristics observed among psychosomatic patients, the term alexithymia refers to a multidimensional personality construct, defined by a set of four characteristics: 1) difficulty in identifying feelings and in distinguishing feelings from bodily sensations of emotional arousal, 2) difficulty in describing and in communicating feelings to others, 3) lack of fantasy and imagination, and 4) an externally oriented style of thinking [25]. The issue about the nature of the construct, i.e., whether it is more a deficit in emotional processing, or a defensive process, has triggered a vivid debate among psychoanalysts [26]. Even if the deficit view has gained support from experimental research [27], other views have suggested moving beyond the debate by giving a teleological sense to the term “defense”. Indeed, according to McDougall [28], these competing theories about the construct are not mutually exclusive: alexithymia is indeed related to deficits in the cognitive representation of emotions but could also be conceptualized as a massive defense against primitive terrors, rather than neurotic conflicts Several studies have reported high rates of alexithymia in drug-dependent individuals [29] and a significant positive association was found between alexithymic traits and craving, the severity of the disorders, and related difficulties [30].

Alexithymia has a complex relationship with various risk factors for the development of SUD related problems (i.e., drug expectations, negative affectivity, insecure attachment, and personality disorders) [29]. Furthermore, the association between immature defensive patterns and alexithymia is well known [12]. In previous studies considering the relationship between alexithymia and defensive array in alcohol dependent patients [23], neurotic and immature defense styles (assessed with DSQ) were higher in the alexithymic group than the non-alexithymic group, and alexithymia positively correlated with the use of passive aggression, acting out, isolation, fantasy, denial, and total immature defense score.

However, it should be noted that the great majority of studies investigating the association between defensive functioning and alexithymia in SUD has relied upon the self-report Defense Style Questionnaire (DSQ) [31]. DSQ, despite methodological challenges in studying unconscious processes by self-report, is the most widely used instrument for defense measurement with validated versions in numerous languages and it showed good reliability and validity [32]. DSQ contains some psychometric shortcomings regarding the overtly pathological wording of the self-report and the unstable factor structure that partially differs from the defensive array described by Bond et al. [31]. To overcome some of the psychometric limitations of the DSQ, the Response Evaluation Measure (REM-71) has been developed [33]. The REM-71 has some advantages when compared with the DSQ. REM-71 items are simpler than DSQ items; they avoid the overtly pathological wording of some DSQ items; phrases involving outcomes (I do X to achive Y) are not used to avoid the confound of dependent and independent variables; REM-71 has a coherent two-factor structure; factor scales are reliable [33]. This questionnaire defines defenses as normative and ubiquitous self-regulatory processes, in line with Vaillant’s model [8].

To the best of our knowledge, no study has been so far conducted using the REM-71 with the specific aim of investigating the relationship between defensive functioning and alexithymia. Therefore, the goal of this study was, first, to investigate potential differences in terms of defensive functioning between SUD patients and non-clinical controls. Secondly, we aimed at investigating the relationships between alexithymia and maladaptive/assimilation defenses (REM-71 Factor 1) in the SUD group. As a last step, we aimed at characterizing in the sample of SUD patients the potential association between defense type (i.e., REM-71 Factor 1: maladaptive/assimilation defenses; Factor 2: adaptive/accommodation defenses) and personality and psychopathological characteristics. We expected that SUD patients would show a more maladaptive defensive functioning compared to that of controls.

Furthermore, we expected that a pattern of maladaptive defenses in the clinical sample would show strong correlations with alexithymia scores, and that an above-cutoff score in REM-71 Factor 1 (4.40) [9] would be associated with impaired psychological well-being, and with difficulties in identifying and describing feelings.

## Methods and procedures

One hundred and seventy-one first-time admitted patients were recruited in nine therapeutic communities in southern Italy, Department of Addiction Services of Bari, National Health Service (ASL BA).

The criteria for inclusion in the study were the presence of a formal diagnosis of SUD (according to the DSM-5), and age above 18. Patients with independent or substance-related current major psychiatric disorders were excluded after a comprehensive psychiatric assessment. Clinical participants had been undergoing their clinical treatment for not less than 1 month and not more than 3 months. One hundred and forty-two (83%) were males and 29 (17%) were females. Their mean age was 36.5 years (SD = ± 8.41).

Among SUD patients, 52 (30.4%) had a diagnosis of Alcohol Use Disorder from moderate to severe, 35 (20.5%) had a diagnosis of Stimulant Use Disorder (cocaine) from moderate to severe, 84 (49.1%) had a diagnosis of Opioid Use Disorder (heroin) from moderate to severe. Ten (5.8%) had a primary school diploma, 99 (57.9%) had a first-level secondary school diploma, 54 (31.6%) had a second-level secondary school diploma, eight (4.7%) had a bachelor degree. Participants were monosubstance-dependent users (with the exception of nicotine, caffeine, and/or past cannabis dependence). They were recruited after the toxicology screening procedure that was independently performed by the therapeutic communities.

One hundred and fifty-five control participants with no history of SUDs were recruited randomly in the population through advertisement. Controls were screened with the Alcohol Use Disorders Identification Test (AUDIT) [34], the Drug Use Disorders Identification Test (DUDIT) [35], and the Italian version of the Structured Clinical Interview for DSM-5 (SCID-5.CV) [36]. Controls with psychiatric disorders were excluded. One hundred and thirty (83.9%) were males and 25 (16.1%) females. Their mean age was 37.6 years (SD = ± 9.94). 6 (3.9%) had a primary school diploma, 74 (47.7%) had a first-level secondary school diploma, 64 (41.3%) had a second-level secondary school diploma, 11 (7.1%) had a bachelor degree. The study was approved by the Institutional Review Board of the Department of Addiction Services, National Health Service (ASL Bari), and it was conducted in accordance with the Declaration of Helsinki.

All subjects provided their written informed consent.

## Assessment instruments

### Response Evaluation Measure-71 (REM-71)

The REM-71 [33] is a self-report, 71-item questionnaire for the assessment of defensive style. It allows the assessment of 21 defenses, each of which is derived from responses to three or four questions. Subjects are asked to rate their endorsement of each item on a 9-point scale. There are versions of the scale for adults, adolescents, and school-aged children both in self and observer-rated (parent) report [37]. The REM-71 has a 2-factor structure across these age groups. Factor 1 (F1) comprises 14 assimilation defenses that distort reality in accordance to expected outcomes, leading to less adaptive functioning (Acting out, Splitting, Displacement, Dissociation, Fantasy, Omnipotence, Passive-aggression, Projection, Repression, Undoing, Conversion, Somatization, Withdrawal, and Denial). Factor 2 (F2), by contrast, includes 7 accommodation defenses that attenuate unwelcome reality, allowing more adaptive functioning (Sublimation, Humour, Idealization, Intellectualization, Reactive Formation, Suppression, and Altruism) [32]. The terms “assimilation”, (absorbing external information based on one’s internal schema), and “accommodation” (modifying internal schemas based on external information), represent less ambiguous labels to define an adaptive hierarchy as “immature” and “mature,” respectively.

The validation study of the Italian version of the instrument, indicated that both high-order scales showed an acceptable internal consistency [32]. In this study, the mean Cronbach’s α value was .54. Two defenses, Sublimation (α = .22) and Denial (α = .25), had α values less than .40. After deletion of the item 27 used to assess Denial, α value reached .41. The remaining defenses showed α values ranging from .41 (*Reactive Formation*) to .78 (*Withdrawal*).

### Minnesota Multiphasic Personality Inventory-2

The MMPI-2 [38] consists of 567 self-report items, which are rated by the participant as true or false. Scores are summarized into 9 validity scales and 10 clinical scales; a number of additional content and supplementary scales can also be scored. Raw scores are converted to T scores (M = 50, SD = 10) relative to normative data. Scores of 65 or above are considered to be in the clinically significant range.

### Toronto Alexithymia Scale (TAS-20)

Alexithymia was assessed with the Italian versione of TAS-20 [39]. The self-report questionnaire is comprised of 20 items rated on 5-point Likert scales ranging from 1 (strongly disagree) to 5 (strongly agree). The TAS-20 consists of 3 factors: difficulty identifying feelings (*DIF*); difficulty describing feelings (*DDF*); externally oriented cognitive style of thinking (*EOT*).

The Italian version of the TAS–20 showed good internal consistency (Cronbach’s α of .75 and .82 in normal and clinical groups, respectively) and high test–retest reliability over 2 weeks (r = .86). A confirmatory factor analysis revealed the same factor structure as the original English version and adequate internal consistency of the subscales, with α coefficients equal or greater than 70 [39].

### Analyses

The statistical pckage SPSS (Chicago, IL) 24.0 for Windows was used for all the analyses.

In order to investigate the defensive array in SUD patients compared to that of non-clinical controls, we compared the mean scores of the four groups (i.e., Control, Alcohol Use Disorder, Stimulant Use Disorder, Opioid Use Disorder) performing a series of univariate ANOVAs. A contrast analysis was run in case of a significant F test and the level of significance was corrected according to Bonferroni procedure for multiple contrasts (*p* = 0.05/4 = 0.0125). We used Student t test for indipendent samples to compare clinical characteristics between the SUD group and the control group. To investigate the role of single defences in explaining the TAS-20 subscores a series of stepwise multiple regression analyses was carried out with the *DIF, DDF, and EOT* as dependent variables and the socio-demographics characteristics of participants (gender, age, and years of education), and the REM-71 defences as predictors.

We compared the personality and psychopatological mean scores (MMPI II clinical and content scales, alexithymia) amog the SUD subgroups (i.e. Alcohol Use Disorder, Stimulant Use Disorder, Opioid Use Disorder) trough a series of univariate ANOVAs with Bonferroni correction.

For all statistical analyses, *p* values were considered significant at *p* < .05.

## Results

### Socio-demographic variables

No significant differences between SUD and control group were found in socio-demographic variables. In the control group, no significant differences regarding socio-demographic variables were found in MMPI II, REM-71, and TAS-20 scores except for the higher female score on REM-71 *Somatization* (*p* < .05).

In the SUD group, gender differences in several scores were found. Female participants showed significant higher scores on MMPI II Validity *F* scale (*p* < .001); on Clinical scales *1 - Hs* (p < .001), *2 - D* (*p* < .001), *3 – Hy* (*p* < .01), *4 - Pd* (*p* < .01), *6 - Pa* (*p* < .001), *7 - Pt* (*p* < .05), *8 - Sc* (*p* < .001), *9 - Ma* (*p* < .05); on Content scales *Anxiety* (p < .001), *Obsessiveness* (*p* < .01), *Depression* (*p* < .001), *Health Concerns* (*p* < .001), *Bizarre Mentation* (*p* < .001), *Cynicism* (*p* < .05), *Low Self-Esteem* (*p* < .01), *Family Problems* (*p* < .01), *Work Interference* (*p* < .05), *Negative Treatment Indicators* (p < .05); on TAS-20 total score (*p* < .05); on REM-71 *Factor 1* (*p* < .01), *Acting out* (*p* < .001), *Splitting* (*p* < .05), *Displacement* (*p* < .01), *Dissociation* (*p* < .05), *Fantasy* (*p* < .01), *Projection* (*p* < .01), *Repression* (*p* < .05), *Somatization* (*p* < .001), *Altruism* (*p* < .05). Among clinical participants, female participants showed significant lower scores on REM-71 *Intellectualization* (*p* < .05). MMPI II clinical scales abbreviations are explained in Table 1.

**Table 1:** Descriptive statistics for REM-71, MMPI II, TAS-20 scores both in the SUD and control groups

		SUD group (*n* = 171)	Control group (*n* = 155)	t (*d.f.* = 324)	*p* value	Cohen’s *d*
REM-71 FACTOR 1 defenses	Acting out	5.03 ± 2.21	3.77 ± 1.93	5.415	**p < .001**	.61
	Splitting	6.45 ± 1.75	6.39 ± 1.72	0.338	NS	–
	Displacement	4.01 ± 2.21	4.03 ± 2.06	−.076	NS	–
	Dissociation	3.79 ± 1.83	3.41 ± 1.69	1.924	NS	–
	Fantasy	4.75 ± 2.03	3.84 ± 1.92	4.169	**p < .001**	.46
	Omnipotence	5.88 ± 1.75	5.41 ± 1.93	2.314	**p < .05**	.25
	Pass.-aggression	5.20 ± 2.00	5.41 ± 1.93	1.293	NS	–
	Projection	3.67 ± 2.09	2.87 ± 1.66	3.786	**p < .001**	.42
	Repression	4.77 ± 1.98	4.91 ± 1.94	1.615	NS	–
	Undoing	5.38 ± 1.98	4.76 ± 1.74	2.878	**p < .01**	.33
	Conversion	2.03 ± 1.53	1.91 ± 1.47	.707	NS	–
	Somatization	3.79 ± 2.21	4.02 ± 2.15	−.968	NS	–
	Withdrawal	6.19 ± 2.17	5.88 ± 2.17	1.293	NS	–
	Denial	5.25 ± 1.73	5.35 ± 1.65	−.550	NS	–
REM-71 FACTOR 2 defenses	Sublimation	5.38 ± 1.98	4.93 ± 1.41	4.935	**p < .001**	.26
	Humour	4.85 ± 1.89	5.02 ± 1.97	−.780	NS	–
	Idealization	5.94 ± 2.06	6.09 ± 1.91	−.675	NS	–
	Intellectualization	5.22 ± 1.75	5.63 ± 1.72	−2.108	**p < .05**	.24
	React. Formation	4.47 ± 1.76	4.42 ± 1.63	.277	NS	–
	Suppression	5.20 ± 1.72	5.13 ± 1.72	.409	NS	–
	Altruism	7.27 ± 1.42	7.11 ± 1.59	1.008	NS	–
	FACTOR 1	4.76 ± 1.24	4.32 ± 1.03	3.475	**p < .001**	.39
	FACTOR 2	5.46 ± .97	5.53 ± 1.00	−.688	NS	–
MMPI II Validity scales	L	49.78 ± 8.13	47.42 ± 9.74	−1.713	NS	–
	F	66.71 ± 18.11	54.08 ± 12.57	5.060	**p < .001**	.81
	K	41.11 ± 10.52	42.92 ± 7.82	−1.235	NS	–
MMPI II Clinical scales	1 Hs - Hypochondriasis	55.25 ± 12..29	53.33 ± 10.52	1.099	NS	–
	2 D - Depression	60.90 ± 12.98	50.95 ± 9.56	5.523	**p < .001**	.87
	3 Hy - Hysteria	55.13 ± 11.73	48.18 ± 7.55	4.318	**p < .001**	.70
	4 Pd - Psychopathic Deviate	67.82 ± 11.90	52.78 ± 8.40	9.152	**p < .001**	1.46
	5 Mf - Masculinity/Femininity	51.41 ± 11.09	48.65 ± 8.46	1.752	NS	–
	6 Pa - Paranoia	65.02 ± 15.72	52.52 ± 9.85	5.836	**p < .001**	.96
	7 Pt - Psychasthenia	58.36 ± 12.31	48.93 ± 10.65	5.379	**p < .001**	.82
	8 Sc - Schizophrenia	62.46 ± 16.52	49.76 ± 10.55	5.628	**p < .001**	.92
	9 Ma- Hypomania	61.51 ± 13.76	55.23 ± 12.12	3.200	**p < .001**	.48
	0 Si - Social Introversion	55.48 ± 12.79	52.30 ± 9.19	1.797	NS	–
MMPI II Content scales	ANX - Anxiety	61.74 ± 20.08	56.53 ± 8.79	1.954	NS	–
	FRS - Fears	55.18 ± 12.49	53.02 ± 7.77	1.269	NS	–
	OBS - Obsessiveness	58.93 ± 13.18	53.35 ± 10.89	2.998	**p < .01**	.46
	DEP - Depression	65.83 ± 14.93	53.33 ± 8.77	6.184	**p < .001**	1.02
	HEA - Health Concerns	61.16 ± 13.46	55.40 ± 11.38	3.020	**p < .01**	.46
	BIZ - Bizarre Mentation	62.77 ± 17.94	55.53 ± 9.46	3.004	**p < .01**	.50
	ANG - Anger	60.49 ± 12.92	56.36 ± 11.92	2.219	**p < .05**	.33
	CYN - Cynicism	57.98 ± 9.84	57.63 ± 11.56	.234	NS	–
	ASP - Antisocial Practices	62.37 ± 11.40	58.23 ± 11.04	2.497	**p < .05**	.37
	TPA - Type A	55.18 ± 10.99	54.42 ± 11.04	.477	NS	–
	LSE - Low Self-Esteem	59.67 ± 13.75	52.12 ± 8.47	4.038	**p < .001**	.40
	SOD - Social Discomfort	53.80 ± 12.06	50.95 ± 10.12	1.671	NS	–
	FAM - Family Problems	62.03 ± 12.74	53.60 ± 10.27	4.703	**p < .001**	.73
	WRK - Work Interference	61.06 ± 13.83	54.33 ± 10.22	3.500	**p < .001**	.55
	TRT - Negative Treatment Ind.	60.37 ± 12.50	56.12 ± 10.13	2.417	**p < .05**	.37
TAS-20	Total score	54.10 ± 12.67	45.95 ± 9.48	6.529	**p < .001**	.73

### Differences between SUD and control group

Descriptive statistics from the Student t test comparing REM-71, MMPI II, TAS-20 mean scores between the SUD and the control group are summarized in Table 1.

As shown, when considering the SUD group, we found a significantly higher score for 5 out of 14 Factor 1 defenses than that of the control group: *Acting out* (*p* < .001); *Fantasy* (*p* < .001); *Omnipotence* (*p* < .05); *Projection* (*p* < .001); *Undoing* (*p* < .01). These results also reflect in Factor 1 total score, wich was higher in the SUD group (*p* < .001). Among Factor 2 defenses, no differences were found except for the higher score on *Sublimation* in the SUD group (*p* < .001)*,* and *Intellectualization* in the control group (*p* < .05).

Taking into account the differences on REM-71 scores among the SUD subgroups and the control group (Table 2), Alcohol Use Disorder patients showed an higher mean score on 5 Factor 1 defenses: *Acting out* (*p* < .001); *Fantasy* (*p* < .001); *Dissociation* (*p* < .05)*; Projection* (*p* < .001)*; Undoing* (*p* < .01). When considering Stimulant and Opioid Use Disorder patients compared to controls, we found higher scores for 3 defenses: *Acting Out* (*p* < .001); *Fantasy* (*p* < .001); *Sublimation* (*p* < .001).

**Table 2:** Descriptive statistics for REM-71 scores for each of the 4 groups, univariate ANOVAS and contrast analyses

	Alcohol Use Disorder group (*n* = 52)	Stimulant Use Disorder group (*n* = 35)	Opioid Use Disorder group (*n* = 84)	Control group (*n* = 155)	F (3,325)	p value	Partial *η*^*2*^
REM-71 FACTOR 1 defenses							
Acting out	5.44 ± 2.20*	5.11 ± 2.01*	4.75 ± 2.26*	3.78 ± 1.93	11.026	**p < .001**	.093
Splitting	6.78 ± 1.70	6.39 ± 1.65	6.28 ± 1.82	6.39 ± 1.72	.902	NS	–
Displacement	4.42 ± 2.09	3.91 ± 2.43	3.79 ± 2.14	4.03 ± 2.06	1.044	NS	–
Dissociation	4.33 ± 1.96 *	3.44 ± 1.54	3.61 ± 1.80	3.41 ± 1.69	3.666	**p < .05**	.033
Fantasy	4.19 ± 1.97*	4.86 ± 1.96*	4.61 ± 2.11*	3.84 ± 1.92	6.066	**p < .001**	.053
Omnipotence	5.83 ± 1.85	6.17 ± 1.47	5.80 ± 1.73	5.41 ± 1.93	2.135	NS	–
Pass.-aggression	5.24 ± 1.90	5.03 ± 2.03	5.24 ± 2.07	4.93 ± 1.64	2.298	NS	–
Projection	4.45 ± 2,23*^,s,o^	3.11 ± 1.70	3.42 ± 2.03	2.87 ± 1.66	9.577	**p < .001**	.082
Repression	4.98 ± 1.98	4.87 ± 2.03	4.59 ± 1.97	4.41 ± 1.94	1.353	NS	–
Undoing	5.76 ± 1.97*	5.27 ± 1.99	5.20 ± 1.99	4.76 ± 1.94	3.706	**p < .01**	.033
Conversion	2.53 ± 1.83 ^o^	1.98 ± 2.55	1.73 ± 1.23	1.91 ± 1.47	3.272	**p < .05**	.030
Somatization	4.25 ± 2.43	3.47 ± 1.01	3.64 ± 2.12	4.02 ± 2.15	1.482	NS	–
Withdrawal	6.55 ± 2.37	6.16 ± 2.22	5.98 ± 2.02	5.88 ± 2.17	1.301	NS	–
Denial	5.52 ± 1.75	5.11 ± 1.82	5.13 ± 1.68	5.35 ± 1.65	.775	NS	–
REM-71 FACTOR 2 defenses							
Sublimation	5.74 ± 1.85*	5.79 ± 1.33*	5.72 ± 1.46*	4.93 ± 1.41	8.088	**p < .001**	.070
Humour	4.99 ± 2.01	4.48 ± 1.68	4.92 ± 1.89	5.02 ± 1.97	.761	NS	–
Idealization	6.12 ± 1.89	6.15 ± 2.13	574 ± 2.12	6.01 ± 1.92	.781	NS	–
Intellectualization	4.90 ± 1.84	5.18 ± 1.48	5.44 ± 1.79	5.63 ± 1.72	2.519	NS	–
React. Formation	4.76 ± 1.89	4.36 ± 1.78	4.34 ± 1.68	4.42 ± 1.63	.757	NS	–
Suppression	5.08 ± 1.74	5.03 ± 1.94	5.35 ± 1.62	5.13 ± 1.72	.473	NS	–
Altruism	7.33 ± 1.53	7.39 ± 1.27	7.19 ± 1.42	7.11 ± 1.59	.511	NS	–
FACTOR 1	5.09 ± 1.21*	4.68 ± 1.19	4.60 ± 1.25	4.32 ± 1.03	6.165	**p < .001**	.054
FACTOR 2	5.53 ± .97	5.39 ± 1.00	5.44 ± .97	5.53 ± 1.00	.313	NS	–

*Indicates the significance of contrasts opposing SUD subgroups patients to controls

^s^ Indicates the significance of contrasts opposing Alcohol Disorder patients to Stimulant Use Disorder patients

^o^ Indicates the significance of contrasts opposing Alcohol Disorder patients to Opioid Use Disorder patients

Furthermore, we compared the SUD participants who scored above the cut-off (i.e. F1 mean score of 4.40 or higher) to the SUD participants who did not. Participants with a pattern of maladaptive/ assimilation defenses (*n* = 99, 57.6%) showed significant higher mean scores on all the personality and psychopathologic variables assessed in the study (*p* < .001), except for MMPI-II scales *3 - Hy* and *5 - Mf* (data not shown).

### Differences between SUD groups

When considering personality and psychopathological traits among the SUD subgroups, Alcohol Use Disorder patients showed a depressive profile compared to Stimulant Use Disorder patients, with higher scores on MMPI II *2 - D* (*p* < .05), *DEP* (p < .05), *LSE* (*p* < .05), and *SOD* (*p* < .001). In addition, Stimulant Use Disorder patients showed higher scores on MMPI II *TPA* (*p* < .05) compared to Opioid Use Disorder patients. Among the SUD subgroups, no differences were found in alexithymia scores.

### Correlation and regression analysis

Finally, in order to investigate relations between MMPI-II Clinical and Content Scales scores, level of alexithymia, and the use of assimilation/accomodation defenses in the SUD group, the Pearson’s r correlation analysis was permormed. The results are summarized in Table 3.

**Table 3:** Analysis of correlations between personality and psychological variables, and REM-71 Factor 1/Factor 2 score (SUD group)

	Factor 1 score	Factor 2 score
	*r* - Pearson	*r* - Pearson
Alexithymia - total score	.714***	.257**
TAS-20 - Difficulty describing emotions	.515***	.163*
TAS-20 - Difficulty identifying feelings	.747***	.228**
TAS-20 - Externally oriented style of thinking	.338***	.202**
MMPI-II		
1 Hs - Hypochondriasis	.377***	−.059
2 D - Depression	.356***	−.058
3 Hy - Hysteria	.101	−.231**
4 Pd - Psychopathic Deviation	.323***	−.057
5 Mf - Masculinity/Femininity	.012	−.024
6 Pa - Paranoia	.520***	.138
7 Pt - Psychasthenia	.545***	.131
8 Sc - Schizophrenia	.589***	.189*
9 Ma- Hypomania	.490***	.389***
0 Si - Social Introversion	.505***	.048
ANX - Anxiety	.636***	.149
FRS - Fears	.468***	.146
OBS - Obsessiveness	.648***	.311***
DEP - Depression	.577***	.131
HEA - Health Concerns	.601***	.171
BIZ - Bizarre Mentation	.620***	.300***
ANG - Anger	.612***	.238**
CYN - Cynicism	.607***	.362***
ASP - Antisocial Practices	.477***	.332***
TPA - Type A	.484***	.296***
LSE - Low Self-Esteem	.602***	.299*
SOD - Social Discomfort	.454***	.069
FAM - Family Problems	.495***	.088
WRK - Work Interference	.665***	.246**
TRT - Negative Treatment Ind.	.649***	.341***

*p < .05; **p < .01; ***p < .001

TAS-20 *– Difficulty identifying feelings (DIF)* showed a strong correlation (r = .747, *p* < .001) with Factor 1 mean score. To investigate the role of single defences on difficulty identifying feelings, a stepwise regression analyses was carried out with the *DIF* subscore as dependent variable and the socio-demographics characteristics of participants (gender, age, and years of education), and the REM-71 defences as predictors. Five defences showed significant associations with *DIF*: the greater the use of *Projection* (38% of variance explained, β = .270, t = 4.232, *p* < .001), *Fantasy* (R2 Change = .13, β = .247, t = 4.224, p < .001), *Acting out* (R2 Change = .05, β = .158, t = 2.251, *p* < .05), *Repression* (R2 Change = .02, β = .176, t = 2.690, *p* < .01), and *Dissociation* (R2 Change = .01, β = .159, t = 2.438, *p* < .05), the more individuals had difficulty identifying feelings [F(1,164) = 5.943, Durbin-Watson = 1.84, *p* < .05]. The final regression model explained the 58% of *DIF* variance (*p* < .05). In the control group, *DIF* showed a moderate correlation (r = .360, *p* < .001) with Factor 1 mean score. Furthermore, we carried out a series of stepwise multiple regression analyses to shed light on the relationship between the other TAS-20 subscores and the Factor 1 defenses. The *DDF* subscore showed a significant correlation (r = .515, *p* < .001) with Factor 1 defenses. In our SUD sample *DDF* was mainly explained by *Repression* at 28% (β = .324, t = 3.979, *p* < .001), to which *Withdrawal* and *Acting Out* added an additional 4 and 2%, respectively, of explained variance after controlling for sociodemographic variables. The final model explained 34% of the variance (F [1.166] = 6.07, *p* < .001). The *EOT* subscore showed a moderate correlation (r = .338, *p* < .001) with Factor 1 mean score. *EOT* was mainly explained by *Displacement* at 9% (β = .202, t = 2.328, *p* < .05) to which *Projection* added a small account of explained variance (2%). The final model explained 11% of the variance (F [1.167] = 4.83, p < .05).

## Discussion

In this study we assessed defensive functioning in SUD patients using the REM-71. The first aim of the study was to investigate the defensive functioning in SUD patients compared to the defensive functioning of non-clinical controls.

Our results support the hypothesis that SUD patients show a more maladaptive defensive pattern than that of non-clinical subjects. When considering the SUD group, we found a significantly higher scores for 5 out of 14 Factor 1 defenses than that of the control group (*Acting out, Fantasy, Omnipotence*, *Projection, Undoing)*. Among Factor 2 defenses, no differences were found except for the higher score on *Intellectualization* in the control group and higher *Sublimation* scores in the clinical sample. SUD patients with a F1 score above the clinical threshold showed a worse psychological functioning in all the domains explored in the study, compared to SUD patients with a F1 score under the cut-off.

When compared to controls, Stimulants and Opioids Use Disorder patients shared the same defense pattern characterized by the use of *Acting out*, *Fantasy*, and *Sublimation*. No differences in the MMPI (with the exception of *TPA* higher scores in Stimulants Use Disorder patients), and alexithymia scores where found between Stimulants and Opioids Use Disorder patients. In the only previous study using the DSQ with opioids dependents [40], heroin dependent inpatients were using immature defense mechanisms (particularly acting out and splitting) than the controls.

In our study, Alcohol Use Disorder (AUD) patients showed a more maladaptive array of defense, including *Acting out, Fantasy, Omnipotence, Projection, Undoing.* These results are in line with previous studies [5, 23]. Because immature defenses generally block conscious awareness of distressing material, operating a rigid and excessive distortion or reality rather than allowing the individual to consciously acknowledge it [41], using substances may be a chemical way of coping with problems. AUD and SUDs patients showed higher levels in *Sublimation.* Notably, the use of sublimation among addicted patients is not so rare. In a study conducted in Turkey, sublimation, pseudoaltruism, acting-out, isolation and autistic fantasy discriminated substance dependents from healthy controls [22]. Grebot and Dardard [42] also found a significant association between the intensity of cannabis addiction and the use of sublimation. In a study by Abd Halim and Farhana Sabri [43], sublimation was a prominent defense in relapsing addicts. Sublimation is a constructive defense employed to deal with unacceptable thoughts, emotions, and impulses, by channeling them into positive and socially acceptable behaviors. The urge to use potentially lethal drugs is an expression of hostility towards those around the addicted person. Family members cry and worry that they will find their loved one dead. There is something wrong with our finding that drug use represents a “sublimation”. Drug use is not a “positive and socially acceptable behavior”. Therefore, we must call our finding of more “sublimation” in drug users over controls a construct error in the research design. It is probably impossible to use drugs addictively while employing a mature defense such as sublimation that makes loving those around one at least partially conscious. Therefore, our finding could be a meaningless artefact probably due to the low α value of the REM-71 *Sublimation* domain (α = .22).

In our study, with regard to personality and psychopatological differences among SUD sub-groups, AUD patients showed a pattern of depressive symptoms when compared to Stimulant Use Disorder patients. Epidemiological data suggest that the linkages between the AUD and major depression (MD) cannot be accounted for fully by common factors that influence both disorders, and that the disorders appear to be linked in a causal manner. According to metanalytic data [44], the most plausible causal association between AUD and MD is one in which AUD increases the risk of MD. Potential mechanisms underlying these causal linkages include neurophysiological and metabolic changes resulting from exposure to alcohol. Furthermore, the use of maladaptive defenses among dependent patients might be the consequence of alcohol dependency during the active phase of disorder. The capacity to use adaptive defenses may diminish in the acute phase of the disorder and AUD patients may use more maladaptive defenses (acting out, projection, dissociation, splitting). During sobriety period, as the depressive and anxious symptoms remitted, their defensive functioning may return to a higher level of maturity [45].

Future longitudinal studies should investigate the changes in defense functioning during detoxification.

No significant gender differences were found in the use of defenses among control participants, with the exception of higher scores on REM-71 *Somatization*. Female participants tend to deal with emotional conflicts or internal/external stressors by the expression of psychological conflict via bodily symptoms without symbolic content. This finding is in line with the previous research that observed somatization as far more common in women [46–48]. Research findings showed that women tend to use more internalizing defenses (i.e. somatization), while men tend to use more externalizing defenses (i.e. acting out), in line with theoretical and clinical observations [49]. However, some studies on gender difference in defense style have provided conflicting findings [32], pointing out the role of cross-cultural factors reinforcing or prohibiting the use of certain defenses via socialization patterns [50]. In the SUD group, there were important gender differences in the frequency of use of Factor 1 defenses and altruism. Watson and Sinha [50] found that female subjects are more likely to use altruism, consistent with the results of our study.

The defenses endorsed more strongly by female participants were *Acting out*, *Splitting, Displacement*, *Dissociation*, *Fantasy*, *Projection*, *Repression*, and *Somatization*.

In our study, we found strong correlations between TAS-20 scores and the use of maladaptive/assimilation defenses. Additional to the previous studies with the DSQ we found the TAS-20-*DIF* to be the main alexithymia factor related to Factor 1 defenses. Several studies suggest that individuals with higher DIF scores, conversely with respect to EOT, showed higher psychiatric symptoms and distress [51]. Consistently, Ueno et al. [52] conducted a cluster analysis on healthy individuals and found two distinct alexithymia subtypes characterized, respectively, by higher scores in DIF and neuroticism, and by higher scores in EOT and lower openness to experience. Moreover, Chen and colleagues [53] found that individuals with introversive-high-alexithymia (characterized by higher scores in DIF and DDF, and lower scores in EOT) tended to show fewer effective strategies to regulate their emotions.

A regression analysis showed that Projection, a psychological defense mechanism proposed by Anna Freud (1937) in which an individual attributes unwanted thoughts, feelings and motives onto another person, explained the 38% of the variance of DIF, after controlling for gender, age, and years of education. In our study, DDF was mainly predicted by Repression at 28%, whereas EOT was independently predicted by Displacement (9%), and it was weakly associated to maladaptive/assimilation defenses. Focusing on the association between Factor 1 defenses and alexithymia, it is noteworthy that DDF and EOT mean scores were explained by more hysterical/neurotic (Repression and Displacement) defenses, according to the Vaillant hierarchical classification [8]. On the other hand, DIF was mainly associated with Projection, a defense characterized by disavowal of reality. Therefore, individuals with higher impairments in the ability of identifying feelings, and in the modulation of affective processes via cognitive strategies, tend to apply maladaptive strategies (including more maladaptive defenses) for the regulation of affect in stressful situations [54].

In SUD group, Factor 1 and Factor 2 scores shared positive correlations with 10 out of 29 clinical variables. All the statistically significant Factor 2 correlations had values going from small to moderate effect size. Factor 2 scores did not correlate (or showed small negative correlations) with most of the clinical scales of MMPI-II (with the exception of Schizophrenia and Hypomania), and with the content scales designed to assess anxiety and depression. Factor 1 scores showed positive correlations with 27 out of 29 clinical variables with moderate to strong effect size. These results were generally supportive of the expected relationships between maladaptive/assimilation defenses and psychopathological distress across several domain. On the other hand, Factor 2 in our clinical sample showed less informative results and did not clearly correlate with a better functioning. These findings need further exploration and research to shed light on the role of Factor 2 defenses in addiction.

There are important limitations to this study. First, defense style was assessed through self-report instruments. Criticism has been raised against the use of self-report for the assessment of defensive functioning [55]. Future studies might address this issue by comparing the REM-71 scores with clinical ratings, with the Thematic Apperception Test (TAT) [56] according to Cramer’s instructions [57], and with performance-based tests (i.e., Rorschach R-PAS [58]). Furthermore, two defenses, *Sublimation* (α = .22) and *Denial* (α = .25), had α values less than .40. These findings suggest a need for a reconsideration of this two REM-71 subscales in the future.

The cross-sectional design of the study does not allow any inference on the causal relationship between defenses and the other psychological variables assessed in this study. Prospective and longitudinal studies, according to Bond suggestion [59], would be the ideal study to find correlations between preexisting defenses and specific illness.

Despite these limitations, our findings suggest that SUD patients are using maladaptive/assimilation defense styles more, and that assimilation defense patterns are related to alexithymia, particularly the *DIF* factor, and to a worse psychological functioning.

Furthermore, these findings provide support of the adoption of the REM-71 as a useful screening instrument among addicted patients.

## Data Availability

The datasets used and/or analysed during the current study are available from the corresponding author on reasonable request.
